# Bibliographical Notices

**Published:** 1855-01

**Authors:** 


### BIBLIOGRAPHICAL NOTICES.

*On the Nature, Signs, and Treatment of Child-bed Fevers, &c.* By Chas. D. Meigs, M. D., Professor of Midwifery, &c., in Jefferson Medical College. Blanchard & Lea, 1854.

This work, addressed by Dr. Meigs, in a series of lively and instructive letters to his pupils of the Jefferson Medical College, has for its avowed object, to prove, that there is no such thing as puerperal fever, that is a primary fever, produced by a vitiated or contaminated condition of the blood, causing, as an effect, the anatomical lesions founded on dissection. This fermentation, putridity, poisonous material, or whatever else it may please physicians to call it, is utterly repudiated by Dr. Meigs as an unworthy remnant of the cerrement de lait doctrine, originating in ignorance and error, but now, by a sound and searching pathology, abandoned to stupid old women, or unprincipled quacks. Dr. Meigs maintains, that puerperal fever is a misnomer, which has helped to mislead and deceive, by fostering a belief that there is a specific cause of the disease independent of, and primary to the local inflammation; his firm conviction after great experience, careful thought, and diligent study of almost all the authors of note upon the subject, is, that all child-bed fevers, (of course he excludes the ephemera,) are primary inflammations of various portions of the intro-pelvic and abdominal regions and that whether that inflammation be a peritonitis, a metritis, a metro-peritonitis, or a metro-phlebitis, there is but one rational and scientific mode of treatment; that treatment is, blood letting, prompt and without stint. He admits that other modes of treatment have succeeded, but looks upon such cases as mere accidents; such patients recovered, but the doctor who prescribed did not cure; he merely stood by and witnessed a great struggle betwixt life and death, in which, happily, life prevailed.

But there are cases, numerous in hospitals, where the patients are in a sinking typhoid condition from the onset; is this puerperal fever? Dr .Meigs says no, but it might be typhus with a superad ded puerperal lesion. But if it be typhus, how is it that a disease, almost universally believed to be contagious, kills all the parturient women, and leaves untouched the old women, the young girls, and the men? Is this a valid argument? By no means. Our author is prepared for every thing, and provides for this difficulty by the somewhat startling assertion, that typhus fever is not contagious. To be sure he qualifies the opinion afterwards by saying, that he is not sure that it is not contagious, but entertains great doubts of it; nevertheless, takes advantage of the doubt, to sustain his own views and opinions, which, as might be anticipated, are totally opposed to the possibility of contagion in any form of puerperal disease. He maintains, that the opinion so general in the community and the medical profession, viz., that infection is often conveyed by nurses and doctors to their parturient patients, is utterly ridiculous. We confess we envy Dr. Meigs this conviction, so earnestly and eloquently expressed, for the doctrine would, if firmly believed in, prove an inestimable blessing and balm to the unfortunate practitioner, who should find himself and his patients the victims of this fearful scourge. To a sensitive nature, so situated, to doubt upon the subject of contagion would be distraction; to believe in it despair. But our author places him at once above such weakness. If patient after patient succumb, while fifty other practitioners never see a case, it is a singular coincidence, but nothing more; he has only to go on hoping and bleeding, and saying, with the gallant Frenchman, "c'est la fortune de la guerre."

The following passage shows how decided the opinions of our author are upon the subject of contagion: "You know that child-bed fever is contagious, because Dr. A. meets in his practice with seventy cases of the disease, while Dr. B., an equally busy man, does not encounter a single one, though they cross each other's path every day. Dr. A.'s track is marked out by victims, while there are no traces of Dr. B.'s path, except it be in recovered women. And you reiterate that facts are stubborn things. Yet you do not know, you only infer and suspect, or surmise that, if Dr. B. had taken charge of A.'s cases, and vice versa, the result would have been the same on the whole, only the dead women would have lived, and the others would have perished. Who told you so? You believe so. Well, I have some small respect for your belief, while I should bow reverently to your knowledge; you believe so, I don't believe so at all."

Well, we cannot say that we do know it for certain; but if such a case as Dr. Meigs proposes were brought forward, we think that the keenest non-contagionist would be staggered by it; he might not, certainly, be fully convinced, but he must doubt. Nor could the fine spun arguments and scientific jargon of ten thousand doctors convince the public mind that such a combination of circumstances was not the result of contagion. True, it may be said by professional men, that the public has no right to be considered in a purely scientific discussion. But this question is no such thing. One set of eminent medical men have proved to their own satisfaction, that puerperal fever is not contagious; another equally learned, eminent, and experienced, maintain that it is; a third party also highly accomplished and respectable, are not willing to commit themselves on either side of the question, but go on doubting, and will doubt until they cease to be, so that all their science put together, is but a jumble of hypotheses.

The following is a quotation from Professor Dubois, whom Dr. Meigs invokes to sustain his doctrine:

"When in an establishment for lying in women, several women are successively attacked, it is impossible to say whether there was infection or contagion in the case."

Again Professor Dubois says:

"But what shall we say of an indirect contagion, of which a healthy person, in some way, serves as the vehicle, and which could not pass from one woman to another, except in this way?

The possibility of such transmissions has been suspected, inferring from facts worthy of attention, doubtless, but the significance and importance of which have been singularly exaggerated, through ignorance, malice, or the spirit of system, the histories of them not being sufficiently circumstantial or precise to carry away the conviction of sober men."

We think that Prof. Dubois, in this instance, makes two perfectly unsupported assertions. In the first place, we have the honest admission of many able practitioners that they believe themselves to have been the means of transmitting contagion to their patients. Dr. Gordon records it of himself in sorrow and tribulation. Now doctors are seldom wilfully malicious to themselves, and it was the interest of these men to ignore the facts if it were possible for them to twist their consciences to such a point.

Again, the histories of these instances of induced disease may not be perfectly convincing; but we affirm, without fear of contradiction, that many of them are as clear and circumstantial in their detail of the following up of one case upon another, and traced from one victim to another, as distinctly as is to be found in the history of any contagious disease that has ever been described. We admit that the deductions therefrom may be erroneous, as they have often been, where the contagion of yellow fever, of plague, and many others has been the subject of argument, but the narrations are as systematic as the very nature of the subject (contagion) will admit of.

One of our author's arguments against the contagion of puerperal fever, in our opinion a very weak one, is as follows:

"It is an absolute condition of contagion, that it must undergo some certain incubation. If it is a ferment, it must have time to ferment; if a spore, it must have time to develope spores, &c."

But are we prepared to assert, that, during a severe epidemic of puerperal fever, some fermentation or incubation may not have been proceeding in the system, which, like a smouldering fire, only needed the stimulus of parturition, to burst into a blaze.

The following extracts show that Dr. M. admits this:

"Die Geburtskunde, &c., of Franz Kiwisch, 11 Abtheil 1 Heft, contains the remarks of that admirable writer on acute blood dissolution, and cholæmia of pregnant and lying in women. He says, it is allied to a diseased state of the blood in gestation, and that it sometimes exhibits its dangerous character in the clearest manner; and as an example of the malady he cites the following curious case, which is that of a woman 22 years old, of a stout well developed frame, and who, on the afternoon, on the 21st of January, 1851, came from the country to the lying-in hospital.

She had hardly left the conveyance and mounted the first step, when she fell, and with difficulty was got into the labor ward. It was her first pregnancy; slightly jaundiced; face cyanotic; extremities cold; pulse scarcely discerned; drops of thin dark blood from nostrils and genetalia; small ecchymoses on skin; could give no account of herself; pains were feeble; os uteri large as a dollar, and tense; head high up; fœtal heart plainly audible. At 10 P. M. she seemed to be sinking, and was then delivered with the forceps, in half an hour after which she expired. The dissection exhibited a tendency within the encephalon, thorax and abdomen, to oozing of blood here and there from the tissues; but there were nowhere any positive marks of inflammation."

It suits the views of Dr. Kiwisch and Meigs to call this a case of blood dissolution or purpura hemorrhagica. This robust, vigorous woman, died with all the worst symptoms of adynamic fever, because her endangium or blood membrane could no longer continue its office of nervous induction into the blood. Why this is a mere evasion; it means nothing, but that Drs. Meigs and Kiwisch cannot see a fever where a puerperal woman is concerned; if no contaminating or disorganizing cause killed that blood membrane, it becomes the vaguest of all possible speculations.

The author then addresses the following words to his pupils:

"I am well pleased to have recited for you Kiwisch's case of acute blood dissolution, because it ought to show you that a pregnant woman may, even before labor begins, have her blood in such a condition as to destroy her suddenly, and also that the blood might become so greatly changed under the irritations which in pregnancy supervene for some women, that there is the highest probability of diseases following the birth of the child."

Yet Dr. Meigs denies that there can be a fermentation or blood poisoning; he insists, that the endangium is the seat of the primary abnormal action, which affects that fluid secondarily; but there are certainly other causes for disease in the blood. Besides the derangement of the doctor's favorite membrane we may cite cholæmia, caused by a derangement of the hepatic functions. And does not urea accumulated in the blood poison to death; or if the lungs lose their power of sufficiently oxygenating the circulatory system, is it not poisoned by carbonic acid? Besides, whether the blood be dependent on the endangium, or that membrane on the blood for its healthy action, or whether both, or neither have any direct or primary influence, is after all but hypothesis. To use the author's favorite expression, we don't know it.

Nor are dissections revealing inflammatory lesions, upon which, of course, the opponents of the existence of puerperal fever support their position, as definite and conclusive as they suppose, for surely no scientific physician, unblinded by some sense-absorbing theory, will maintain that pathology is synonymous with morbid anatomy, or that the scalpel is the only mode of investigating disease.

This question, no doubt, will long continue to be a vexed one. The present state of our knowledge upon the whole subject of contagion is too uncertain and contradictory to form data for settled opinions, and so long as authors, however talented, continue to interpret the phenomena of nature to suit their own peculiar views of a subject, it must remain a fruitful source of contention.

That part of Dr. Meigs work most interesting and instructive, is where he treats of uterine phlebitis, the production of pus and the effects of its entrance into the circulating system. His descriptions of the symptoms attending this fatal accident are perfect, and his instructions for making correct diagnosis most practical and useful.

In letter xv. the progress of a fatal case of metro-phlebitis is as graphic in description as it is touching and eloquent in composition. We cannot resist the pleasure of making the following quotation, though our space is limited:

"What shall I say, in a weak, vain hope of portraying the feelings with which the physician approaches the bedside of one so perilous; what of the deep inner conviction with which, on glancing at the countenance, the decubitus, the gestures, or on touching that hurrying artery, or on perceiving that morbid heat or coolness, or hearing those expressions that lift him on the one hand to the height of hope, or whisperingly tell that the vein is inflaming more and more, that the purulent infection is begun, and that his art, with his wishes, prayers, hopes, are all alike in vain."

The next is a very fine description:

"Then came calm tranquillity, the greatest gentleness, scrupulous politeness, and careful attention to her personal appearance and array, but the pulses ever beat faster and weaker; eructations of gases; then of colorless fluids; then porraceous vomitings; then darker ones; and at last black vomit; lessening pulses; increased respiratory efforts; cooling hands and feet, that turned bluish in their cyanosis, until at last a death-like coldness overspread the limbs, and the icy king advanced slowly to the conquest he made over what was most enchanting among educated, elegant women. Stupor, lethargy, scarcely to be roused, then deep coma, and at last death; rest for the victim, and tears and suppressed moanings for the bereaved. Why say all this? Why, a physician to practice midwifery must be made of stone if he would not feel on these occasions; and if he have a heart of flesh, he surely deserves the sympathy of all good people when he turns away a baffled man, after faithful, well conducted efforts to save one whom no art could save."

Dr. Meigs treats of all the usual remedies in puerperal fever. His directions for their use are given with all the author's acknowledged practical sense and discretion; but bleeding is his sheet anchor, though he admits that cases do occur where it is inadmissible; such he regards as very unpromising, and measures the chances of success by the amount of bleeding which the patient can bear. Now the whole profession, we think, will agree with him in admitting that in all those cases, accompanied by frank, febrile symptoms, such treatment is excellent, and will often effect a cure. But Dr. Meigs avoids mentioning a fact equally well established, viz., that those puerperal diseases which bear free, sanguineous evacuations are the least formidable.

Though we differ with some of the theories advanced in this book, we sincerely admire its manifold merits, and would be far from wishing to snatch one laurel from the brow of its accomplished author. The epistolary form, we confess, we do not like, and the style of Dr. Meigs is at times wordy even to affectation, but he has evidently studied his subject profoundly, and throws himself into it with an honest fervor which rivets his reader's attention. The earnest intensity and sympathy of many of the paragraphs could only emanate from a gentle, generous spirit.

---

*A Systematic Treatise, Historical, Etiological and Practical, on the Principal Diseases of the Interior Valley of North America, &c.* By DANIEL DRAKE, M. D. *Edited by* S. HANBURY SMITH, M. D., &c., and FRANCIS G. SMITH, M. D. &c. *Second series.* Philadelphia, Lippincott, Grambo & Co. 1854.

This most comprehensive work of the lamented Drake will be received with no common feelings by the medical profession of our country. Its posthumous character will at once recall those personal and intellectual traits which made its author beloved and respected by all who knew him. Dr. Drake was no ordinary man. With few advantages of early education, his untiring industry and devotion to his studies, his enthusiastic and enquiring mind, and his excellent natural abilities, enabled him to amass an amount of knowledge which was truly wonderful. His various and extensive writings show how earnest and severe were his labors in behalf of that science to which he had devoted himself. By these and his public teachings, he did much to stimulate and improve the Western medical mind. He was also a zealous advocate and promoter of every scheme of public improvement. It may be truly said of him that he possessed all the attributes which should belong to the Physician, being able, conscientious and true-hearted. Even now, though he has passed away from us, his life-long testimony remains, a memory and example which can never die.

> "Only the memories of the just
> Smell sweet and blossom in the dust."

The present work, Book Second, comprising nearly one thousand pages, embraces five parts, in which the subjects of Autumnal Fever, Yellow Fever, Typhous Fevers, Eruptive Fevers, and Phlogistic Fevers, (the Phlegmasiæ,) with their histories, modes of action, pathology, treatment, &c., are most fully and accurately described. It is mainly, however, to the immense amount of local information afforded us, information which spreads over a vast extent of country, and to the writer's ingenious speculations, that the work owes its principal value.

The progress of society in the Great Valley is indicated in the following curious passage:

"In the early settlement of the country, the border warfare with the Indians led to many gunshot wounds; while the warlike spirit, which external dangers nourished, assumed the character of pugnacity, and led the hardy and fearless backwoodsmen to turn upon each other. Both casual and pitched battles were common events; but the state of society was so primitive, that the instruments of mischief were generally the hands and teeth. A pommelling with the first, sufficient to give two or more cases of inflammation and fever, was a frequent result; and the injury and loss of an eye, followed by the same consequences, was equally common; while severe bites on the face and hands now and then gave poisoned wounds, which healed with difficulty. The secretion of an acrid saliva under the influence of rage, is an effect which finds its parallel in the carnivorous animals, and cannot, therefore, be rejected as visionary. With the progress of society this pugnacity has signally abated; yet enough remains to render it formidable to life, for the pistol and the bowie knife have in many parts of the Valley supplanted the teeth and fist."

As a book of reference alone, containing matter which it would be difficult, if not impossible, to obtain elsewhere, Dr. Drake's work should command a place in the library of every American physician. The print and paper do great credit to its enterprising publishers.

*The modern treatment of Syphilitic Diseases, both primary and secondary; comprising the treatment of constitutional and confirmed Syphilis by a safe and successful method; with numerous cases, formulæ and clinical observation.* By LANGSTON PARKER, Surgeon to the Queen's Hospital, Birmingham. *From the Third, and entirely re-written edition.* Philadelphia, Blanchard & Lea, 1854.

The volume before us contains very full and perspicuous information on the treatment of Syphilitic Affections, both primary and constitutional. In addition to the extensive experience derived from a large hospital, Dr. Parker states, that he has personally treated more than eight thousand cases. From this ample store he has constructed the present work, which will be found an excellent guide by those who need information on these subjects. He considers the simple non-mercurial treatment as more proper in the primary treatment than in the secondary form of the disease. Even in this form, the cures are often more apparent than real, the disease recurring again when the patient returns to his customary occupation. When low diet, rest, opiate and astringent washes, aperients, &c., have all failed to cure, then, and not till then, he uses mercury, and "this is certainly," he states, "the result of modern experience on the subject." The indications for the use of mercury in primary syphilis are the following:—"When a sore remains long open and shows no disposition to heal under the non-mercurial plan indicated. 2. When secondary symptoms appear before the primary disease is cured. 3. In well marked indurated chancre, more especially if these have been tested by inoculation. 4. In all characteristic sores which have yielded a characteristic pustule by inoculation; the indications for the employment of mercury in the two last mentioned class of cases is still more pressing, if the primary sores be accompanied by bubo. 5. In certain cases of rapidly spreading ulceration, hereafter to be mentioned."

Of the internal administration of mercury *alone*, for the cure of the disease, he has a poor opinion. He entirely agrees with Sir B. Brodie, who says:—"You may patch up the disease by giving the remedy internally, but it will return over and over again." The treatment by inunction, he considers to be both more certain, milder and safer. It is essential, however, that the patient be confined to a warm room, or to his bed. Infantile syphilis is stated to be more successfully treated by this method than by any other. The plan, however, which his experience has satisfied him to be the most successful, and accompanied with the fewest disadvantages, is *fumigation.* This is done by vaporising the mercury from a tin plate by means of a spirit lamp, and mixing it with a small quantity of common steam, so that the patient may be exposed to a gradually increasing temperature. With this method, he advises the administration of small doses of mercury, as, for instance, the twentieth of a grain of the bichloride or biniodide and a milk diet. In the 22nd chapter, full directions are given as to the apparatus required, the form of mercurial, and other requisites needed in fumigation. He considers the treatment by vapor to be applicable to *every* form of constitutional syphilitic disease, and beyond all doubt the most speedy, certain, and safe remedy that can be employed. We shall not enter into any further remarks upon Dr. Parker's work. We shall merely state that it has passed through three editions in England, has long been recognized as a standard work there, and is well and favorably known to the profession in this country. We dislike the wording of the title page. It strikes us as being too much in the vein of the advertising gentry. With this exception, we sincerely recommend the work to our readers.

---

*Nature in Disease, illustrated in various Discourses and Essays, to which are added Miscellaneous Writings, chiefly on Medical Subjects.* By JACOB BIGELOW, M.D., Physician and Lecturer on Clinical Medicine in the Massachusetts General Hospital, etc. Boston: Ticknor & Fields. 1854.

This little volume contains a collection of Dr. Bigelow's papers, chiefly bearing on the subject of "nature in disease," the basis of which was his discourse on "self-limited diseases," delivered before the Massachusetts Medical Society in 1835.

The study of the natural history of disease is closely connected with an accurate study of pathology, and thus it was that the "vis medicatrix naturæ," although so frequently invoked in the writings of the older physicians has not been carefully investigated until the present era in medicine.

In the volume before us Dr. Bigelow enquires into the individual diseases which belong to the class of self-limited diseases, and in how far medicines are capable of controlling morbid changes. Remarks on so important a subject, coming from one who for upwards of a quarter of a century has been such a close observer, must necessarily be listened to with attention by the whole profession.

The chief diseases which are at present considered self-limited, are measles, scarlet fever, and small pox. To these Dr. Bigelow adds of the more common affections, erysipelas and typhoid fever. The latter he considers self-limited mainly on account of its marked affinity with the class of eruptive fevers. We have, however, more than mere analogy in favor of this view. Both Louis and Nathan Smith, names intimately connected with the history of typhoid fever, affirm this to be the result of their long experience.

The great barrier to an appreciation of the effect of remedies must always be the difficulty of distinguishing between the symptoms of diseases and those produced by the use of the medicines employed. There are, besides, other obstacles which blind our judgment, and which are so happily alluded to by Dr. Bigelow, that we will be excused for producing the passage at length.

"Independently of the common defects of medical evidence, our self-interest, our self-esteem, and sometimes even our feelings of humanity, may be arrayed against the truth. It is difficult to view the operations of nature, divested of the interferences of art, so much do our habits and partialities incline us to neglect the former, and to exaggerate the importance of the latter. The mass of medical testimony is always on the side of art. Medical books are prompt to point out the cure of diseases. Medical journals are filled with the crude productions of aspirants to the cure of diseases. Medical schools find it incumbent on them to teach the cure of diseases. The young student goes forth into the world, believing that if he does dot cure diseases, it is his own fault. Yet when a score or two of years have passed over his head, he will come at length to the conviction, that some diseases are controlled by nature alone. He will often pause at the end of a long and anxious attendance, and ask himself, how far the result of the case is different from what it would have been under less officious treatment, than that which he has pursued; how many in the accumulated array of remedies, which have supplanted each other in the patient's chamber, have actually been instrumental in doing him any good."

We might quote still further from this excellent little essay, replete as it is with the sound thoughts of a distinguished medical philosopher, but we have extracted enough to show its general character, and to recommend it most strongly to the attention of every reflecting physician. Besides the discourse on self-limited diseases, Dr. Bigelow's little work contains many interesting papers, of which the Report on Homœopathy, the Remarks on the Mucuna Pruriens, those upon the poisonous effects of the American Partridge, and the Experiments on Pneumothorax, are most worthy of notice.

---

*Phreno-Geology: The Progressive Creation of Man indicated by Natural History, and confirmed by discoveries which connect the organization of the brain with the successive geological periods.* By J. STANLEY GRIMES. Boston and Cambridge: James Munroe & Co. London: Edward T. Whitfield. 1851.

We have been induced to notice the work before us, although not within the legitimate field of our duties, by the fact that it has obtained a large circulation, particularly in the Western States, and is probably considered by many persons ignorant of the subjects touched upon as setting forth orthodox scientific views. We shall make no apology, therefore, for directing attention to a work which would otherwise only merit contempt and silence.

It is mainly owing to such writers as Mr. Grimes that so much dislike exists in many minds to the study of those discoveries of science which seem to be in opposition to the letter or spirit of revelation. We find, indeed, several instances where the *literal* interpretation of scripture *is* at variance with the clearly established facts of science, yet little difficulty need be found in reconciling them, when we bear in mind that the language used must of necessity have been such as could be easily comprehended by the uncultivated minds to whom it was addressed. We may be sure that no *real* discovery *can* be at variance with divine or scientific truth. Our duty is simply to *prove the truth* of the discovery and of the theories founded thereon, and we need not fear that in so doing we are entering upon forbidden ground.

Very different is the course pursued by the promoters of that false philosophy founded upon groundless hypothesis and superficial learning, that finds its best security in attacking the statements of the Bible, and hides the utter want of foundation for its facts and arguments by virtually thrusting forward the proposition, that as the statements of Genesis are not apparently in accordance with some of the discoveries of geology, therefore their discoveries are true, since they contradict the book of Genesis also. To the ignorant and those who cannot obtain better information from books or persons, the false statements and idle theories that are found in works of this class appear true and just, and their minds become filled with errors that shake their faith in the religion of their youth, giving them in place thereof nothing but the nonsensical views of a charlatan.

Others, again, whose religious opinions are deeper rooted, but who are as equally unfit as the former class to judge of the truth of the so-called new discoveries, naturally look at them all in the same light, and avoid troubling their consciences with what they call "the vain learning of the world."

The title that the author of the subject of our notice has chosen, phreno-geology, indicates the nature of the work. It is an endeavor to connect phrenology with the successive creative periods that the earth has passed through, and to show that each period had its corresponding development of intellectual organs in the brains of its inhabitants, which gradually became more and more elevated in their structure as the earth advanced through its changes, until at last the mind of man was the result. He endeavors to establish the following points, viz:

"1st. That the organs of the human brain are added and superadded in a manner such as they would be if they had been successively created to conform to the geological changes which took place after the first animal was created.

"2d. That the convolutions of the brain are arranged as they would be if caused gradually by the pressure of the brain during birth.

"3d. That the pons and the callosum are added to hold the two hemispheres together.

"4th. That the physiognomy of man was created and caused by his habits while he was yet below the standard of modern humanity.

"5th. That instead of the earth being created for the animals which it contains, and adapted to them, man and all other animals have been created by the agency of the infinite variety of stimulating circumstances which have been brought to bear upon organized bodies during the immense periods of time indicated by geology."

The author adopts Lamark's view, that man is the product of a progressive development from the lowest form of animal life into a higher type, the changes being induced by the necessities of the creature and propagated in its descendants. It is needless to attempt a refutation of so absurd a doctrine. Its advocates have utterly failed in adducing a single instance of a transformation among even the lowest types into one more elevated, nor can they even produce a more plausible hypothesis in favor of it than that, because the lower forms of animal life existed before the higher, therefore the lower forms are developed into the higher. With as much propriety might we watch the gradual growth of vegetation upon a rock in its process of disintegration into soil, and exclaim, behold! the oak is but the development of the lichen, for on this rock we found, first, the lichen, then the mosses, next the creeping wood plants, and now, here is the oak.

A few extracts will show the learning, logical skill, modesty, and high religious tone of the author.

"A modification of the theory of Lamark has been brought forward lately in a work entitled the 'Vestiges of Creation.' The truth of this doctrine is, however, denied by many geologists and naturalists, among whom is Mr. Lyell, . . . . . . Cuvier, and most of the great European and American naturalists. But history teaches us to receive the published opinions of popular and salaried philosophers upon such subjects with much allowance for the delicate circumstances in which they find themselves placed. It is dangerous to advocate important truths in advance of the age. Diana of the Ephesians is still too great to be approached without prudence and respect."

The world at large should be grateful that Mr. Grimes's position as a lecturer upon phrenology does not place him within the ranks of trammelled philosophy.

Another of his remarks is the following:

"It is a startling announcement, that our ancestors once inhabited the mighty deep, and, sustained on broad extended fins, roved through the vast ocean. But if our theory is admitted, such is the inevitable tendency of the arguments, and nothing but the intervention of a miracle can prevent this conclusion."

How beautiful Trinculo's description of Caliban meets the above view. "What have we here? a man or a fish? dead or alive? A fish! he smells like a fish; a very ancient and fish-like smell! a kind of, not of the newest poor john; a strange fish!"

The argument (?) principally relied upon appears to be the following:

"By the will of God, the increasing coldness of climate produced the principal circumstances that produced animals and man. The earth was once too hot to allow of the existence of animals; they were not produced until it had cooled down to a certain point. Plants, infusoria, radiates, molusks, trilobites and fishes were then created. I do not mean to assert that these animals were created in the order named; they may have originated and progressed simultaneously. If the earth had continued at the same temperature which it then possessed until the present time, it is certain that man never would have existed in his present form. We should now have all been fishes or nothing,—*aut pisces, aut nullus.* We could not even have advanced to the dignity of reptiles, enjoying the privilege of crawling occasionally out of the water into the mud on swampy islands of the sultry ocean. Still colder must it have been when our great reptile parents left the ocean altogether, stood upon the solid earth, fed upon its herbage, and breathed with lungs instead of gills. It must have been colder, or such an atmosphere could not have existed. I doubt not that the atmosphere was the agent under Providence that created the lungs—the solid earth created the feet—the food created the teeth and digestive organs—the temperature of the air created the skin, hair, and feathers of land animals, and the light created and modified the eyes. It may have been millions of years in doing this, but geology does not restrict us in regard to time. Only admit, as every one must, that some slight change in organization can be produced in a thousand years exposure to some powerful influence, and the whole argument is at once surrendered, for geology instantly steps in with its vast period of time to accomplish any amount of transformation which organization is capable of undergoing without destruction. It should also be considered that organization is capable of gradually assuming any form whatever that can be conceived, provided circumstances require it, and sufficient wholesome food, air, and protection can be obtained by the change, and not otherwise. The point that I am now insisting upon, is, that by the will of God, cold produced the circumstances which created man. Admitting that 'the vile race from whom we sprang' once inhabited the water, it is plain that when primeval man in reptile form first left his native ocean, bade it farewell, and established himself upon the land, feeding exclusively upon its productions, if the temperature of the earth had remained stationary from then until the present time, man in his present form would not have existed." p. 19.

"There is no scientific evidence that a single organic thing on earth was ever created suddenly. Every thing is formed by the aggregation of many atoms, and always under circumstances favorable to such aggregation. The aggregation of chemical atoms formed minerals, *the minerals composed mountains, but were an immense number of years in doing so.* Just so chemical elements combined to form vegetables, and the vegetable organisms, aggregated and arranged in a peculiar manner, compose animals. The first elements that were created, had certain forms adapted to their need and condition; a change in their circumstances produced a change in their forms, until man was produced. To say, then, that man was made at once, in a single day, from dust or chemical atoms, is to make man an exception to all the rest of nature." p. 32.

The only other "*argument*" that can be found, consists in the frequent repetition of the doctrine that "we are not required to believe anything which is absurd or revolting, merely because it is taught by the strict letter of Genesis." p. 34.

The above passages do not deserve serious refutation, being merely groundless assertions and untenable positions; yet many persons, not familiar with the subject, are doubtless misled by such bold and assured statements.

Mr. Grimes is indebted to the author of the "Vestiges of Creation" for the following absurd deduction. He has faithfully preserved the spirit of the original.

"This process is called crystallization. Sometimes the crystals arrange themselves in forms very similar to the forms of vegetation; this fact is often demonstrated in winter mornings upon our windows, in the various fantastic forms which the frozen vapor assumes. The resemblance of these figures to vegetables strikes every observer, *and inclines us to suspect that the same general law operates in both cases.*" p. 45.

"Vegetation seems to be essentially a modification of the principles of crystallization. Certain mineral substances in the earth are held in solution by water, and certain gaseous substances are held in solution by the atmosphere. When a proper degree of heat is applied, the two solutions act upon each other in a way which is not well understood, but the result is a combination of the ingredients of both, which assumes regular forms. *This is vegetation.*" p. 46.

Very clear and lucid; a most valuable addition to our knowledge of vegetable physiology.

The word Magnetism serves the same purpose to the pseudo-philosophers of the present day, that the term "Ether" did to the ancients; that is, whenever they are utterly unable to understand or explain a phenomenon, they connect with it in some manner magnetism or magnetic force, and then rest contented under the delusion that the whole subject is made perfectly clear. Mr. Grimes states, with regard to the origin of mind:

"Mind came into the world when certain organized beings were in peril; it came to save them from death by directing their first feeble movements to the objects which they required. Here stood the plant and there stood the object which it needed, neither could approach the other. If they both floated in air or even in water, we could imagine that some mutual attraction might bring the plant and the nourishing object (food) together on magnetic principles. Perhaps, animal life did commence in such floating circumstances. Perhaps the magnetic principle did operate to bring the plant in contact with its object. . . . . . . My conjecture is, that the action of a species of magnetism between the plant and its food, produced a tendency in the plant to move its parts towards its food, and thus originated muscular motion and animal function."

Mr. Grimes does not appear to consider that his view of the origin of man is even a matter to be questioned. He says:

"It is certain that the ancestors of man and of the ape were once all reptiles, or else that a special miracle of creation has been interposed to prevent it, and to create man suddenly, in a manner which passes human comprehension. . . . . . . . There was certainly a time when reptiles were the highest animals that existed on earth. It is probable that our ancestors were among these reptiles. At that time, the ancestors of all the different races of men and of apes may have been but one species. If the ancestors of the apes left the water, and first became permanent residents on the land, they would immediately assume a character peculiar to themselves. . . . . . . Ages afterwards, another portion may have left the water, and landed on a different shore, and became the ancestors of negroes. Still later and on another island, the ancestors of the white man may have landed; and thus, though all originally were one species, they have been so differently affected by the different circumstances which have operated on them, that they seem to be fundamentally unlike." p. 63.

Mr. Grimes has the highest respect for circumstances; he thinks that they not only "alter cases," but even create legs and feet.

"In regard to the forms of animals, I cannot perceive why an animal may not be of any imaginable form that circumstances may require, for circumstances are the sub-creators of animal forms. *Aqueous circumstances create finny forms of limbs; and airy circumstances create feathers and winged forms of limbs; while terra firma circumstances create feet.*"

Mr. Grimes on Mermaids:

"It may be that the ancient idea of mermaids was not entirely fabulous; and a species of sea mammal as nearly resembling a chimpanzee as the seal resembles the dog, may have but lately become extinct, as many other animals have done. It is to be hoped that the future researches of naturalists may yet throw some light on this obscure question. It would be really delightful, if, in consequence of their labors, we should be able to determine with reasonable precision, all the various forms and changes which our race has undergone, in its rise from the ocean and the mud to the cultivated field and the classic temple. . . . . . . . . . Apes and men may have been alike when they both inhabited the ocean; but the apes degraded themselves beyond redemption by acquiring the habit of walking on their hands, as this circumstance prevented them from acquiring those arts which gave superiority to man, and enabled him to exist in cold regions." p. 143.

Our author's method of reasoning is beautifully shown in this extract:

"It is said, (I know not on what authority,) that the pelican has been known to pierce her own breast, to furnish nourishment for her young. This may have been true, also, of other animals; and if practised by one animal and then by its offspring for several successive generations, this would at length so far modify the constitution of the breast, as to originate the teats and mamma in one female; and from this one, all other mammalia of one species may have descended. This hypothesis is perfectly consistent with admitted principles of Physiology, for it will not now be denied, that the changes produced in one generation are transmitted to the next by hereditary descent." p. 105.

With regard to the difference in the size of animals, he thinks it is wholly dependent upon the amount of nourishment obtainable by the animal.

"If you ask me why the mouse and the ox became thus different in size, I would venture the suggestion that originally all vertebrate animals were of one size, but that some have been, for millions of years, limited in the quantity of nourishment, so that, of course, they were limited in the capacity of receiving functional stimuli; while others have been surrounded with both nutritive material and functional stimuli."

It would be an interesting question to decide whether mice, shut up in a granary, could ever, by such means, be converted into rats; and, also, whether we could reasonably expect to obtain a breed of cattle by a further continuation of the same process.

Mr. Grimes has evidently not much respect for revealed religion; natural religion meets with still less favor at his hands. The principle argument depended upon by theologians, is the perfect adaptation to end manifested throughout creation. He says:

"There is then a continual attrition of every thing by its neighboring things, so that each tends to conform to its surrounding tormentors. The most perfect conformity must always necessarily be produced; this is denominated *adaptation*, and is held up to the ignorant as a sort of miracle, though in reality, it is the necessary consequence of a very simple operation.''

We have not thought it worth while to extract anything from the portion of the work, relating to phrenology. He, there, becomes like nearly all writers upon the subject, utterly wild and nonsensical. Having learnt the anatomical titles for certain portions of the brain, he distributes the pleasures and passions, the virtues and vices among them in a truly phrenological manner. Had he confined himself to that so-called science alone, his book would not have been worth noticing, as the injury it could have done would have been but slight; but by connecting it with the real sciences, he has produced a compound simply nauseating to those acquainted with true philosophy, but poisonous to the young and ignorant.

---

*The Microscopic Anatomy of the Human Body in Health and Disease, illustrated with numerous drawings in color.* By Arthur Hill Hassall, M.B., &c. *With additions to the text and plates, and an introduction containing instructions in microscopic manipulation.* By Henry Vanarsdale, M.D. In two vols. New York: S. L. & W. Wood. 1855.

We expressed a very favorable opinion of this work upon its first appearance, and shall now content ourselves with saying that it is pre-eminently the best illustrated microscopic human anatomy that is accessible to us in this country. The able introduction by the editor, and the numerous and admirable plates added to the American edition render it, in our opinion, far superior to the English one. We consider it, in every respect, a most creditable work to all who were concerned in its publication.

---

*Human Physiology: Designed for Colleges and the higher classes in schools, and for general reading.* By Worthington Hooker, M.D., Professor of the Theory and Practice of Medicine in Yale College, &c. *Illustrated by nearly* 200 *engravings.* New York: Farmer, Bruce & Co. 1854.

The above volume is stated by the author to be designed for the family as well as for the school. He very truly observes that the same clear and full instruction is needed in both instances; the same requirements for information existing in both. Not seeing any reason, therefore, why a physiological work for the people should differ from one written for the school, he has composed his little treatise for the edification of both interests.

The book has THREE PARTS. The first considers organized and unorganized substances; the distinction between animals and plants, and man in his relations to the three kingdoms of nature. The 2d part is devoted to the consideration of the structure of man, his various functions, his formation and repair, and to cell life. The 3d part discusses the nervous system, the bones, the muscles and their language, the voice, the ear, the eye, connection of the mind with the body, differences between man and the inferior animals, varieties of the human race, and life and death.

The subject of "Reproduction" is very properly omitted.

The chief aim of every elementary treatise should be to present a clear and correct outline of the subjects of which it treats. Well established principles only should be inculcated; and the materials by which these are sustained should be judiciously selected. The present work possesses these merits in a very high degree. Like every thing else which has emanated from the pen of Dr. Hooker, it is characterised by sound judgment and good taste. The style, which is that of the lecture room, is clear and agreeable. We take great pleasure in recommending it as a most useful work for the purposes for which it was intended.

---

*The Transactions of the American Medical Association*, Vol. vii. New York: Chas. B. Norton. 1854.

The above contains 200 pages less matter than its predecessor. There are but two illustrations in it, one of which is colored, a map. The present committee of publication must have had a comparatively easy time of it.

We shall take another opportunity of examining its contents.

---

*Diseases and Injuries of Seamen, with remarks on their Enlistment, Naval Hygiene, and the Duties of Medical Officers.* By G. R. B. HORNER, M.D., Surgeon U.S.N., &c., &c. Lippincott, Grambo & Co. 1854.

Several chapters of the above treatise have already appeared in the pages of this journal. To our readers, therefore, it will suffice for us to say that the style and matter of the concluding essays render them in every respect worthy of their predecessors. Though the work is more especially designed for the benefit of those "who are novices on board ship," we are much mistaken if even veteran tars will not be entertained by its perusal.

---

*The Physician's Visiting List, Diary, and Book of Engagements.* Lindsay & Blakiston. 1855.

The experience of the last two or three years has satisfied us that the above is a most valuable *vade mecum.* Physicians will find that it will add greatly to their convenience and advantage to possess a copy of it.

